# Artificial Intelligence in Forensic Sciences: A Systematic Review of Past and Current Applications and Future Perspectives

**DOI:** 10.7759/cureus.70363

**Published:** 2024-09-28

**Authors:** Ioannis Ketsekioulafis, Giorgos Filandrianos, Konstantinos Katsos, Konstantinos Thomas, Chara Spiliopoulou, Giorgos Stamou, Emmanouil I Sakelliadis

**Affiliations:** 1 Department of Forensic Medicine and Toxicology, National and Kapodistrian University of Athens School of Medicine, Athens, GRC; 2 Artificial Intelligence and Learning Systems Laboratory, School of Electrical and Computer Engineering, National Technical University of Athens, Athens, GRC

**Keywords:** artificial intelligence, deep learning, forensic medicine, forensic pathology, forensic sciences, machine learning

## Abstract

The aim of this study is to review the available knowledge concerning the use of artificial Intelligence (AI) in general in different areas of Forensic Sciences from human identification to postmortem interval estimation and the estimation of different causes of death. This paper aims to emphasize the different uses of AI, especially in Forensic Medicine, and elucidate its technical part. This will be achieved through an explanation of different technologies that have been so far employed and through new ideas that may contribute as a first step to the adoption of new practices and to the development of new technologies.

A systematic literature search was performed in accordance with the Preferred Reported Items for Systematic Reviews and Meta-Analyses (PRISMA) guidelines in the PubMed Database and Cochrane Central Library. Neither time nor regional constrictions were adopted, and all the included papers were written in English. Terms used were MACHINE AND LEARNING AND FORENSIC AND PATHOLOGY and ARTIFICIAL AND INTELIGENCE AND FORENSIC AND PATHOLOGY. Quality control was performed using the Joanna Briggs Institute critical appraisal tools.

A search of 224 articles was performed. Seven more articles were extracted from the references of the initial selection. After excluding all non-relevant articles, the remaining 45 articles were thoroughly reviewed through the whole text. A final number of 33 papers were identified as relevant to the subject, in accordance with the criteria previously established.

It must be clear that AI is not meant to replace forensic experts but to assist them in their everyday work life.

## Introduction and background

Forensic Pathology (FP) is meant to aid in the correct and prompt administration of justice. Societies in general, not just the judicial system, profit from an efficient and reliable medicolegal system. A properly functioning medicolegal system supports crime detection and investigation and presents certified expert witnesses in court, thus preventing crime (by establishing means of detection and punishment) and accidents (such as substance abuse or road traffic accidents). FP is a medical specialization that straddles the lines of both medicine and law [1]. This is the reason why FP followed all the medical and technological innovations through the years from Hippocrates to modern laboratories and from the Industrial to the Digital Revolution. Quite soon after the beginning of the latter, in 1956, John McCarthy first described the term artificial intelligence (AI). AI refers to the use of technology and computers to simulate intelligent behavior and critical reasoning that is comparable to that of a human being [2]. The evolution of this idea introduced the term “Machine Learning” (ML), as a technique by which a computer can learn from data without using a complex set of different rules. This approach is based on training a model from datasets. In 1986, Rina Dechter introduced the term “Deep Learning” (DL), as a subcategory of ML. In fact, DL is considered a technique to perform ML inspired by the human brain’s neuron network [3]. Finally, the term “artificial neural network” (ANN) was officially introduced in 2000 by Igor Aizenberg and colleagues [4].

An ANN is a massively parallel combination of simple processing units that can learn from their surroundings and store the information learned within its connections [5]. ANNs are a type of computational model inspired by the structure and functioning of biological neural networks, such as the human brain, and are a fundamental concept in the field of ML. At their core, ANNs are composed of interconnected artificial neurons (also known as nodes or units) organized into layers [6]. It is an umbrella term that incorporates most of the models included in this systematic review.

Convolutional neural networks (CNNs), a class of ANNs, are a type of DL model specifically designed for analyzing visual data, such as images or videos. They consist of multiple layers of interconnected neurons, including convolutional layers, pooling layers, and fully connected layers [7]. CNNs leverage the concept of convolution, which involves applying filters to input data to extract meaningful features and capture spatial relationships. Through a process of hierarchical feature extraction and representation learning, CNNs can automatically learn discriminative features from raw input images [8]. The convolutional layers enable local connectivity and weight sharing, reducing the number of parameters and allowing CNNs to efficiently handle large-scale datasets. Due to their "full connectivity," these networks are vulnerable to data overfitting [9]. CNNs can classify, multi-classify, and multi-label images [10]. CNNs have been extensively employed in various tasks within the domain of Forensic Pathology. 

Backpropagation neural networks (BPNNs) are a type of ANN that uses the backpropagation algorithm for training. Backpropagation is one of the most widely used algorithms for training feedforward neural networks, particularly multilayer perceptrons (MLPs) [11,12].

Robust object detection frameworks (RODFs) refer to a set of algorithms, techniques, and methodologies used to accurately and reliably detect objects in images under various challenging conditions, such as occlusion, variation in lighting, scale, pose, and cluttered backgrounds. The goal of a robust object detection framework is to achieve accurate and consistent detection performance across diverse real-world scenarios [13].

The k-nearest neighbor (k-NN) algorithm is a simple and popular supervised machine learning algorithm employed both for classification and regression tasks [14]. It operates based on the principle that similar data points tend to have similar labels or values. Given a new data point, the algorithm will place it in the multi-dimensional plain where all other known data exist and predict its class or value by looking at the k nearest known data points.

Facing the AI technological revolution, FP is called upon to investigate possible ways to exploit the new technologies at hand. This development represents a whole new chapter in medicolegal sciences that could probably assist forensic pathologists in many everyday challenges. For example, external postmortem findings (e.g. injuries, medical manipulations, etc.) or postmortem imaging findings could be recognized through image processing software that employs AI technology. On the one hand, DL, and AI in general, could lead to the automation of procedures that are time-consuming and accurately provide answers to many questions that today could not be easily answered. On the other hand, the authors are very clear that AI is not meant to replace forensic pathologists, but rather to assist them with data handling and processing. 

To the best of the authors’ knowledge, there is only one similar systematic review concerning forensic sciences, in general. In this paper by Galante et al., the authors attempted to demonstrate most AI technologies that have been used in different forensic fields, in general, by simple quotation without attempting any comparison [15]. 

The aim of this systematic review is to review the available knowledge concerning AI use in different FP areas from human identification (HI) to postmortem interval (PMI) estimation and the estimation of different causes of death (COD). The current study aims to emphasize the different uses of AI, especially in FP, and to elucidate the technical part of AI in this field. This will be performed through an explanation of the different technologies that have been so far used and through new ideas that may contribute as a first step to the adoption of new practices and to the development of new technologies.

## Review

Materials and methods

A systematic literature search was performed in accordance with the PRISMA (Preferred Reported Items for Systematic Reviews and Meta-Analyses) guidelines to increase comprehensiveness and transparency of reporting [16]. Published studies were found using a thorough search strategy of the PubMed Database and Cochrane Central Library. There were neither time nor regional constrictions in our search and all the included papers were written in English language. The terms used in the search were MACHINE AND LEARNING AND FORENSIC AND PATHOLOGY and ARTIFICIAL AND INTELIGENCE AND FORENSIC AND PATHOLOGY. References within the included articles were reviewed and the corresponding abstracts and full articles were accessed in case they were relevant. Studies citing the included articles were searched through the PubMed Database and their corresponding abstracts and full articles were also accessed if relevant. The literature search was performed on February 01, 2024, when this project first took shape, and was held up to April 09, 2024. The aim of this study is to create a database for the use of AI and ML methods that have been tested so far in FP, which could be used as a first step for the development of new algorithms capable of assisting forensic pathologists in their everyday tasks. Research articles without data related to AI and ML algorithms, without data related to FP and/or with data that did not focus on humans, were excluded. The definition of our keywords in the search is as follows. As human, we considered every human being regardless of gender, age, and other population characteristics. Using technology and computers to mimic intelligent behavior and critical thinking that is similar to that of a human being is known as AI, while ML is a method that allows computers to learn from data without the need for a complicated system of principles [2]. Finally, the term Forensic Pathology covers every element of Forensic Medicine that is currently practiced, starting with the medical CODs, certification of cause and manner of death, and autopsy laws, among other things [1]. The information that was abstracted is mentioned below. The abstracted data include author(s), publication year, region of the study, the aim of the study, modality, algorithm architecture, results, limits, and recommendations of the study. Due to qualitative and summative nature of this review and significant variations in study design and reporting, a meta-analysis and statistical calculations were not reported. Forensic terminology is explained in Table 1, while AI models’ terminology is explained in Table 2.

**Table 1:** Forensic Terminology

Term	Description
Postmortem interval (PMI)	The interval between time of death and time of postmortem examination. It can be estimated through postmortem changes on the deceased’s body, environmental conditions at the scene of death, and information on the deceased’s habits.
Human identification	The process of identifying a deceased’s body. It can be accomplished using various methods, mostly through fingerprints, DNA analysis, or panoramic radiographs.
Sex estimation	The assessment of sex from skeletal remains. It can be accomplished through morphological (e.g., the subpubic angle of the pelvis, ramus flexure in the mandible, etc.) or osteometric techniques.
Age estimation	Assessment of age from bone examination. It can be applied to both the living and the dead through examination of bone growth and dental development.
Maturity development estimation	Age estimation that can be accomplished through examination of dental development.
Diatom test	Diatoms are eukaryotic unicellular or colonial algae, which are detectable in water, air, and soil. The diatom test is based on the assumption that diatoms reach the lung with the inhalation of liquid and disseminate through the bloodstream to closed organs if cardiovascular activity exists. This test can be used for the diagnosis of drowning.
Heat-exposed bone	When a bone is exposed to heat, several changes occur that can be used as indicators of the fire temperature, duration, and combustion circumstances. These changes refer mostly to color changes and other morphological changes (e.g., heat-induced fractures, heat-induced dimensional changes).
Pericardial effusion	The accumulation of excess fluid in the pericardial sac that surrounds the heart.

**Table 2:** AI Models' Terminology

Term	Description
Artificial neural network (ANN)	A computational model inspired by the structure and function of the human brain. It consists of interconnected nodes (neurons) that process and transmit information.
Multilayer perceptron (MLP)	A type of feedforward neural network with multiple layers of neurons, including input, hidden, and output layers. It is widely used for various tasks, including classification and regression.
Backpropagation neural network (BPNN)	A type of neural network that uses the backpropagation algorithm for training. It adjusts the network’s weights to minimize the difference between predicted and actual outputs.
Robust object detection framework (RODF)	A set of algorithms, techniques, and methodologies used to accurately and reliably detect objects in images under various challenging conditions, such as occlusion, variation in lighting, scale, pose, and cluttered backgrounds.
k-nearest neighbors (k-NN)	A non-parametric classification algorithm that assigns a class label to a data point based on the majority class among its k nearest neighbors in the feature space.
Convolutional neural network (CNN)	A specialized type of neural network designed for image recognition and processing tasks. It uses convolutional layers to automatically learn hierarchical patterns and features from images.
Supervised learning (SL)	A machine learning paradigm where the model is trained on labeled data, with input-output pairs, to learn a mapping from inputs to desired outputs.
Clustering (CL)	An unsupervised learning technique where data points are grouped into clusters based on similarity. It helps discover hidden patterns and structures within data without labeled examples.

Results

**Figure 1:**
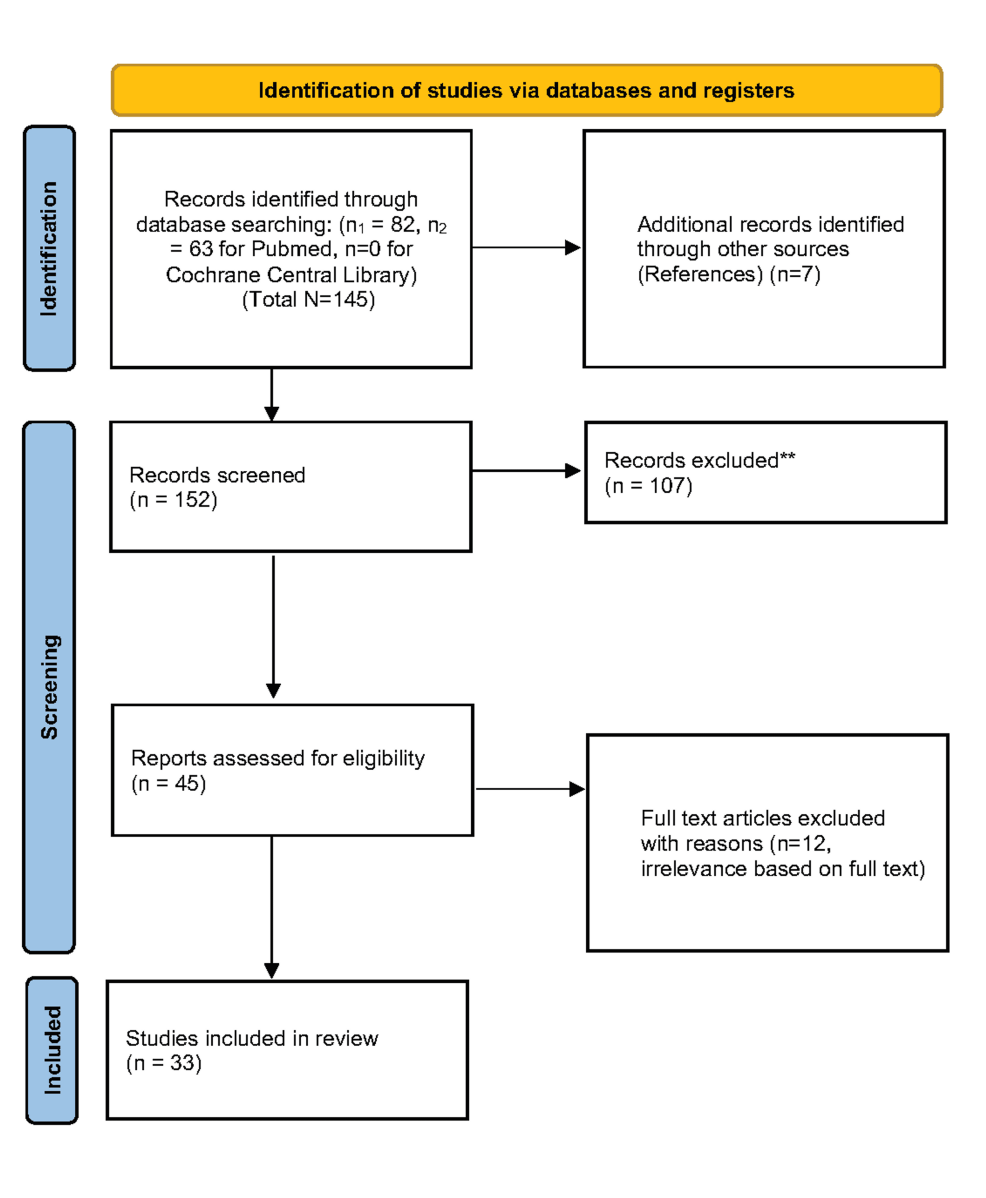
PRISMA Flowchart. PRISMA: Preferred Reported Items for Systematic Reviews and Meta-Analyses

A search of 224 articles, from the PubMed and Cochrane Central Library databases was performed. It is important to highlight the fact that the Cochrane Central Library database did not contain any articles meeting the above-described criteria. In addition, seven more articles were extracted from the references of the initial selection of articles, that were to be accounted for in the literature review. All the above-mentioned articles were thoroughly studied. A total number of 145 articles were checked, through their title and abstract. After excluding all non-relevant articles, the remaining 45 articles were thoroughly reviewed through the whole text. After the whole text study was concluded, a final number of 33 papers were identified as relevant to the subject, in accordance with the criteria previously established. Finally, quality control was performed using the Joanna Briggs Institute (JBI) critical appraisal tools. Using these tools, no articles were excluded. Thus, a total number of 33 papers were included in the current systematic review. All the critical appraisal methods were conducted by two reviewers independently. A PRISMA flow diagram detailing the systematic search is presented in Figure 1. 

The results, summed up in Table 3, present the following study characteristics: aim, modality, algorithm architecture, outcomes, limitations, and recommendations. 

**Table 3:** Summary of Studies Applying Machine Learning in Forensic Medicine and Pathology.

Author	Region	Year	Aim of the Study	Modality	Algorithm Architecture	Results	Limits and Recommendations
Navega et al. [17]	Portugal	2018	Age estimation	Femoral bone mineral density obtained through DXA	ANN (DXAGE)	DXAGE appears to forecast death age as well as the majority of traditional methods for determining age in human skeletal remains	Limited to females of European origin
Li et al. [18]	China	2018	Age estimation	Pelvic X-ray	Fine-tuned convolutional neural network (CNN)	Even for samples from people aged 19, 20, and 21, the CNN can handle all possible cases of automated skeletal bone age assessment	(i) It may not see practical application in determining ages over 22 years and (ii) less accurate than deep learning architectures based on hand X-ray radiographic images
Garland et al. [19]	Australia	2020	Identifying fatal head injuries	Postmortem computed tomography (PMCT)	Convolutional neural network (CNN)	Training dataset accuracy of 92.5% and testing dataset accuracy of 70%	(i) having only 50 cases, (ii) using only one transverse image from the head PMCT scan as the input, and (iii) grouping all different head injury diagnoses as one single group
Lin et al. [20]	China	2019	Determination of causes of death	pulmonary edema fluid samples	Convolutional neural network (DeepIR)	(i) prediction accuracies ranging from 0.8774 to 0.9167 - outperformed all the machine learning-based classifiers, (ii) can easily be optimized to achieve better accuracy, (iii) can be constracted quickly and (iv) able to learn rapidly and effectively from massive amounts of spectral data	involving only five causes of death: sudden cardiac death, drowning, toxication, brain injury and asphyxiation
Mohammad et al. [21]	Malaysia	2022	Segment the maturity development of the mandibular premolars - Dental staging	Panoramic radiographs	Keras-based deep learning convolutional neural networks (DCNN)	(i) training accuracy: 97.74, validation accuracy: 96.63, and testing accuracy: 78.13% and (ii) Although moderate agreement (Kappa value = 0.58) was achieved, no sign of the model’s over-or under-fitting upon the learning process was seen	(i) In order to select a better model with better performance, the methods used to optimize the DCNN model can be extended by adding some additional hyperparameters and (ii) In order to select a better model with better performance, the methods used to optimize the DCNN model can be extended by adding some additional hyperparameters
Boedi et al. [22]	Belgium	2019	Age estimation	Panoramic radiographs	CNN (DenseNet201)	Utilizing a DenseNet201 CNN increased the previously reported staging accuracy	Correct stage allocation was obscured by remaining surrounding tissues
Yu et al. [23]	China	2020	Identify the deaths from drowning	Diatom test	A robust object detection framework (RetinaNet)	(i) average precision: 0.82 and average recall: 0.88, with a threshold of 0.5 (ii) possibly taken into account as a part of the diatom test's automated process	Limited data set
Zhou et al. [24]	China	2019	Identify the deaths from drowning	Diatom test	CNN	(i) accuracy greater than 90%, (ii) the CNN model has learned specific morphological hallmarks of diatoms instead of unwanted background clutter and (iii) more rapid than the traditional microscopic examination	Needs hardware upgrade and larger number of training samples
Wärmländer et al. [25]	Sweden	2018	Estimating the temperature of heat-exposed bone	Portable X-ray fluorescence (pXRF) and spectrophotometer	linear model and the k-nearest neighbor (k-NN) machine-learning algorithm	Spectrophotometric color measurements combined with machine learning methods can be a viable tool for estimating bone heating temperature	(i) Limited sample size, (ii) factors such as burning time and the amount of soft tissue present may affect the color and (iii) screening for chemical contamination may be needed
Wilder-Smith et al. [26]	Switzerland	2022	Detection, segmentation, and classification of pericardial effusions	Chest CT	Deep convolutional neural network (nnU-Net)	(i) PEF detection sensitivity 97% (95% CI 91.48–99.38%) and specificity 100.00% (95% CI 96.38–100.00%), (ii) Diagnosing hemopericardium sensitivity 89.74% and specificity 83.61% (AUC 0.944, 95% CI 0.904–0.984), (ii) model and corresponding datasets are publicly available and (iv) highly robust in different protocols, institutions and patient groups	(i) Trained on the latest generation CTs from a single scanner manufacturer at a single institution, (ii) a larger case number is needed and (iii) inter-reader variability was worse compared to reference-prediction variability
Li et al. [27]	China	2019	PMI estimation	Human annual cartilage samples	FTIR spectroscopy	Cartilage could be considered an ideal matrix for PMI estimation (slower degradation)	Need: (i) Larger number of samples, (ii) vector machine support and (iii) random forest and deep learning
Cantürk and Ozyılmaz [28]	Turkey	2018	PMI estimation	Eye images	Two feature selection algorithms (LASSO and Relief), two classification algorithms (k-NN and LibSVM) and two validation methods (10-fold and LOSO)	(i) very practical (ii) does not need expertise and (iii) can be made available to forensic experts even as a mobile application	Further studies are needed for verification of the method
Garland et al. [10]	Australia	2020	PMI estimation	Postmortem gross images of visceral organs	CNN	(i) the overall accuracies were >95% for both training and testing datasets and (ii) F1 score of >0.95 for all dissected organs	(i) identification of more organs is needed and (ii) identification of organs dissected at different planes and by using different dissection methods is needed
Toneva et al. [29]	Bulgaria	2020	Sex estimation	CT scans	Three data mining algorithms: the rule induction algorithms JRIP and Ridor, and the decision tree algorithm J48. Two advanced attribute selection methods: Weka BestFirst and Weka GeneticSearch	(i) all JRIP and Ridor sets of rules included less than 10 cranial measurements, and only the J48 decision trees used a greater number, (ii) the accuracy of all models is nearly 90%, (iii) the JRIP rule set generated on the GeneticSearch selection dataset is 91.9 %, (iv) Generated rules are easy to apply without the need for any calculation and (v) standard ando nonstandard measurements can contribute to the correct sex estimation based on the human cranium	NS
Zhang et al. [30]	US	2019	PMI estimation	Postmortem microbiomes collected by swabbing five anatomical areas, sequenced and analyzed	xgboost, neural network and random forest	(i) the xgboost method accuracy (74.5%– 87.6%), neural network accuracy (70.7–83.0%) and random forest accuracy (73.6–86.3%) and (ii) random forest was often comparable to xgboost, but could underperform both competing algorithms in specific instances (e.g. PMI > 48 hours)	More studies are needed to develop machine learning guided molecular autopsies
Johnson et al. [31]	US	2016	PMI estimation	Postmortem microbiomes collected by swabbing different anatomical areas	k-nearest- neighbor regressor	(i) predicts the PMI of unknown samples with an average error of ±55 accumulated degree days (ADD) and (ii) useful over a longer period of decomposition time, than this previously described method	(i) multi-site study is now needed to examine the role of local environment and (ii) more data are needed from a large-scale study that will involve several swab sites
Fan et al. [32]	China	2020	Human identification	Panoramic radiographs	CNN (DENT-net)	(i) Rank-1 accuracy: 85.16%, Rank-5 accuracy: 97.74%, (ii) high accuracy and speed and (iii) it can be used without any special equipment or knowledge to generate the candidate images	(i) DENT-net was not yet optimized for human identification on mixed dentition, (ii) some detail identification rules that draw manual attention cannot be learned by the present system and (iii) application in cadavers still must be evaluated
Porto et al. [33]	Brazil	2020	Sex and age estimation	Face photos	artificial neural network classifier	(i) For the sex estimation of individuals over 14 years old, accuracy values higher than 0.85 by the F1 measure, (ii) accuracy 0.72 for the F1 measure with an age interval of 5 years, and (iii) For the age group estimation, the F1 measures of accuracy are higher than 0.93 and 0.83 for thresholds of 14 and 18 years, respectively	(i) non-standardized (frontal) recording of the human face, (ii) need to include data for ages older than the cutoff, and (iii) Need to try to get results from other countries and ethnicities
Liu et al. [34]	US	2018	Pericardial effusion localization and segmentation	CT scans	Cascaded Coarse-to-fine CNN	(i) accuracies are likely to be acceptable for clinical use and (ii) this coarse-to-fine approach can be applied to segmentations of other organs on medical images	NS
Yang et al. [35]	China	2019	Sex estimation	Whole-skull CT scans	backpropagation neural network (BPNN)	(i) Accuracy rate of the training stage: 97.232%, mean squared error (MSE): 0.01 and (ii) Compared with traditional methods, it has stronger learning ability, faster convergence speed, and higher classification accuracy	Larger sample is needed to build a model that could assess sex from unknown bones
Liu et al. [36]	China	2020	PMI estimation	Microbial community characterization and microbiome sequencing from different organs (i.e. brain, heart and cecum)	random forest (RF), support vector machine (SVM) and artificial neural network (ANN)	(i) The ANN model combined with the postmortem microbial data set from the cecum was the best combination with a mean absolute error of 1.5 +/- 0.8 h within 24-h decomposition and 14.5 +/- 4.4 h within 15-day decomposition and (ii) reliable and accurate technology in PMI estimation	Need for a larger sampling time frame
Kahaki et al. [37]	Malaysia	2019	Age estimation	Orthopantomography	deep convolutional neural networks (DCNNs)	The technique can accurately and precisely classify the images with good performance, enabling automated age estimation	(i) Larger data sets are needed with elderly patients and (ii) anisotropic filtering in the early stages of CNN
Ebert et al. [38]	Switzerland	2017	Detection of hemorrhagic pericardial effusion	Postmortem computed tomography (PMCT)	deep learning image analysis software (ViDi Suite 2.0)	(i) two separate deep learning networks, one to classify images into hemopericardium/not hemopericardium, and one to segment the blood content and (ii) The best performing classification network classified all cases of hemopericardium from the validation images correctly with only a few false positives	Underestimates the amount of blood in the pericardium
Zeng et al. [39]	Japan	2023	Diagnosis of fatal hypothermia	Postmortem computed tomography (PMCT)	deep convolutional neural networks (DCNNs)	They used the area under the receiver operating characteristic curve (AUC) of the system for evaluation, and a human-expert comparable AUC value of 0.905, sensitivity of 0.948, and specificity of 0.741 were achieved.	ensemble multiple classifiers to reduce the number of false positives produced by any individual feature. Also, by adjusting the threshold of classification probabilities, the trade-off between sensitivity and specificity can be optimized based on the specific application.
Cheng et al. [40]	USA	2024	human gunshot wound classification	digital color images (jpeg format) of entrance and exit GSWs	A ConvNext Tiny deep learning model	The model achieved an accuracy of 87.99%, precision of 83.99%, recall of 87.71%, and F1-score 85.81% on the holdout set. Correctly classified were 88.19% of entrance wounds and 87.71% of exit wounds.	Image collection and preparation is very time-consuming
Dani [41]	Hungary	2023	Time of Death Estimation	Henssge’s formula data	An SVM with a radial basis function (RBF) kernel and AdaBoost+SVR	estimated time of death accuracy of approximately +/-20 min or =/-9.6 min, respectively, depending on the SVM parameters. The error in the predicted time (tp[h]) was tp =/- 0.7 h with a 94.45% confidence interval	Further testing with real data is needed.

Out of the 33 selected studies, seven were systematic reviews concerning AI use in more specific fields of forensic interest (e.g. Forensic Anthropology, Forensic Odontology). These studies were excluded from the table, as because of their nature (review articles), they did not present any new AI algorithm. Information was collected from regions all around the world including Portugal, Bulgaria, China, Australia, Scandinavian countries, and America.

Subcategorization by the Different Fields of FP

The 26 studies included in this systematic review could be subcategorized by the FP field they address. Forensic Anthropology (FA) is the field of 9 out of 26 studies. Age and sex estimation are the subject of four studies [18,22,37,42] and two [29,35] studies, respectively, while one paper introduced an algorithm able to estimate both [33].

One study aimed to aid in HI through use of panoramic radiographs [32] and another presented a Keras-based deep learning convolutional neural network (DCNN) that could segment the maturity development of the mandibular premolars [21].

Ten out of 23 studies introduced algorithms that could assist in the estimation of the COD. One study identified head injuries that could be fatal [19], while two papers presented an algorithm that facilitated the automation of the diatom test, to be used for drowning identification [23,24]. 

Regarding more specific subjects, one study is relevant to the estimation of the temperature of heat-exposed bones [25], while another one explores fatal hypothermia diagnosis [39]. Three studies aim to assist in the detection, the segmentation, and/or the classification of pericardial effusions [26,34,38]. One study introduces a CNN that could identify five specific CODs [20], while another one employs AI in wound ballistics [40]. Moreover, six studies present algorithms that could assist in PMI [19,25,27,28,31,38]. Finally, in the study by Dani et al. a new approach was proposed to determine the time of death through a support vector machine with a radial basis function kernel and adaptive boosting (AdaBoost) and support vector registration [41].

**Figure 2:**
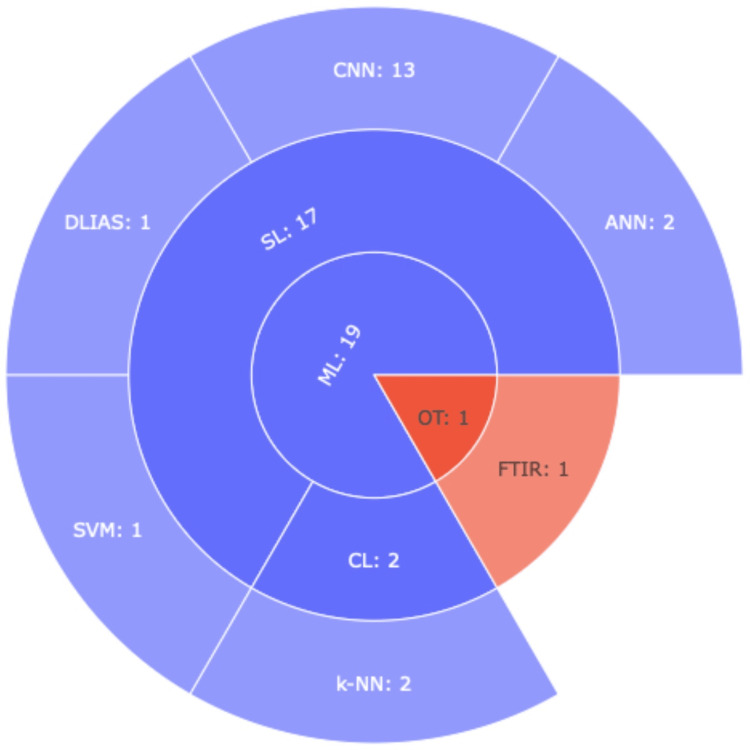
Hierarchical Clustering of Machine Learning Algorithms Analyzed in This Study. The Sunburst Diagram Represents the Hierarchical Clustering of Machine Learning Algorithms. ML: Machine Learning; SL: Supervised Learning, CL: Clustering; CNN: Convolutional Neural Network; ANN: Artificial Neural Network; BPNN: Backpropagation Neural Network; k-NN: k-Nearest Neighbor; RODF: Robust Object Detection Framework; DLIAS: Deep Learning Image Analysis Software; OT: Other Techniques; FTIR: Fourier Transform Infrared Spectroscopy. Each algorithm is categorized based on its specific role within the machine learning domain, showcasing their hierarchical relationships.

**Figure 3:**
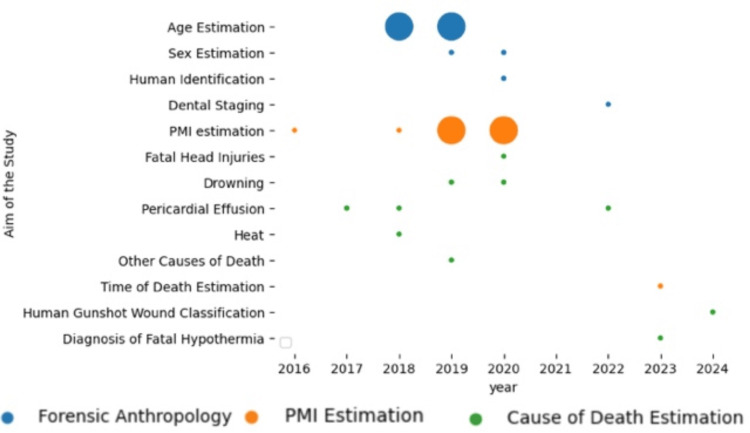
Research Aims Over Time. The horizontal axis represents the years, the vertical axis represents the specific aims of the studies, and each data point on the graph corresponds to the number of papers focusing on a particular aim during a given year. The colors in the diagram indicate the general categories of the tasks addressed in the papers. PMI: Postmortem interval

A hierarchical clustering of the algorithms is depicted in Figure 2, while an illustration of the distribution of research aims over time is depicted in Figure 3.

*Subcategorization by Algorithm Architectures* 

As demonstrated in Table 3, different studies introduced different algorithm architectures. Analytically, eleven studies built CNNs [10,18-22,24,26,29,32,34,37,39,40]. ANNs were provided by two studies [17,33]. One study employed a BPNN [11], one a robust object detection framework [23], and another a deep learning image analysis software [38]. One applied Fourier transform infrared (FTIR) spectroscopy [27], in which the authors used conventional learning methods to establish predicted models, and two were based on k-NNs [25,32]. Finally, four studies applied more than one algorithmic architecture [28-30,36].

Convolutional neural networks: Li et al. presented a deep learning bone age assessment model based on pelvic radiographs for forensic age estimation [18]. The model employed in this study consisted of a pretrained AlexNet architecture, which was utilized for feature extraction [43,44]. Subsequently, three fully connected layers were incorporated into the model, with sizes of 2048, 1024, and 1, respectively. This model was compared with an existing cubic regression model. The results demonstrated that the deep learning model achieved similar performance to the existing model. Similarly, Li et al. developed a deep learning CNN model for forensic age estimation based on pelvic radiographs [18]. Their model was trained on a dataset of 1,875 pelvic X-ray images from individuals aged between 10 and 25 years. They compared the performance of their deep learning model to an existing cubic regression model based on ossification staging methods and found that the DL model achieved performance on par with the existing model, with mean absolute error and root-mean-squared error indicating comparable predictive ability. Furthermore, Garland et al. [19] employed a CNN consist of sequential convolutional layers with filters of 32, 64, 128, and 128, utilizing a kernel size of 3x3 and rectified linear unit (ReLU [45]) as the activation function, accompanied by corresponding max-pooling and dropout layers for distinguishing fatal head injury cases from non-injury controls. The two output layers were configured with 256 and 2 units respectively, employing ReLU as the initial activation function and softmax as the final activation function. 

Regarding the CODs, Lin et al. employed AI to analyze pulmonary edema fluid from forensic autopsies using infrared spectroscopy combined with a CNN [20]. The researchers compared the performance of a CNN model named DeepIR [46] with eight popular machine learning algorithms (SVM, Linear Discriminant Analysis-LDA, etc.). DeepIR utilizes a CNN with an "Inception" module, which consists of four parallel network structures with different convolutional and pooling layers. This module is claimed to enable the extraction of both low and high-level features from one-dimensional spectroscopic data without introducing additional computational complexity, thereby enhancing the network’s width and depth. Mohammad et al. proposed a combination of image processing and machine learning techniques to segment the maturity development of mandibular premolars [21]. For this task, a DCNN [47] model with 3x3 convolutional layers with 64 nodes and two dense layers was utilized. Beodi et al. [22] aimed to improve the automated tooth development staging in subadults by segmenting only the lower third molar in panoramic radiographs, using a DenseNet201 CNN [13]. Yu et al. presented a deep learning-based approach for diatom search automation in scanning electron microscopic images, aiming to improve the time-consuming process of the forensic diatom test [23]. RetinaNet, a CNN-based one-stage object detection framework, was employed for this task. The core of the system was a ResNet-101 [48] containing 101 computational layers and leveraged transfer learning by the model trained on ImageNet [49]. For the same task, Zhou et al. employed a model consisting of five convolution nodes combined with 2 max pooling layers, followed by 11 stacks of inception modules and a fully connected layer for the prediction [24]. Wilder-Smith et al. utilized an Encoder-Decoder architecture CNN [50] for Automated Detection, Segmentation, and Classification of Pericardial Effusions on chest computed tomography (CT) [26]. Garland et al. [10] employed a pre-trained CNN base using Xception, a CNN 71 layers deep [51], swapped with an output layer that consisted of two dense fully connected layers of 256 and 11 (number of classification) units for the classification of gross postmortem images of dissected organs. Fan et al. presented an automatic HI system named DENT-net, based on a customized CNN [32]. The DENT-net model utilized a convolutional neural network architecture with four convolutional layers, followed by max pooling layers and three fully connected layers for identification. The network employed small 3x3 filters and a maximum of 128 filters in each layer. The second fully connected layer with 512 channels. Liu et al. [34] proposed a two-stage method using convolutional neural networks (hybrid neural network-HNN [52] and U-Net [50]) for localizing and segmenting pericardial effusions in CT scans. Kahaki et al. developed an age assessment method utilizing global fuzzy segmentation, local feature extraction, and a DCNN [37]. The proposed architecture consists of multiple layers, including convolutional, rectifier, normalization, pooling, fully connected, dropout, and softmax layers, which collectively perform input processing, feature extraction, and classification with cross-entropy loss, resulting in a classification output with four classes.

Artificial neural networks: Two of the studies [17,33] included in this systematic review employed such networks. In particular, Navega et al. [17] proposed an ANN model based on a MLP [53], using one dense (fully connected) layer with 128 neurons with the Adamax optimizer [54], to automatically extract anthropological information, such as sex and age, from facial images. Porto et al. [33] employed a modified general regression neural network [55], an ANN that attempts to mimic the associative memory, to model the bone mineral density variables, into predictors of age at death.

Backpropagation neural network: Yang et al. trained a feedforward BPNN, developed in MATLAB, to determine gender from 267 skull CT scans reaching 96% accuracy [35].

Robust object detection framework: Yu et al. employed a robust object detection CNN framework called RetinaNet for automatic diatom detection in electron microscopic images [23].

k-nearest neighbors: Wärmländer et al. utilized k-NNs to determine the maximum heating temperatures of burnt bones by their specular component included (SCI) color values [25]. Johnson et al. constructed a k-NN regression, trained with a dataset from nasal and ear microbiome samples, that predicts the postmortem interval (PMI) of unknown samples to within 55 accumulated degree days, or two days at an average temperature of 27.5°C [31].

Discussion

The present systematic review aimed to review the available knowledge of the literature according to the applications of AI in different areas of forensic interest. 

Sex and age estimation is a subject of particular interest, whenever the condition of the corpse does not allow proper identification. Various studies demonstrated that different bony parts of the human body could be employed for age estimation purposes. So far, two CNNs [18,22] and one ANN [17], which can estimate age from pelvic x-rays, panoramic radiographs, and femoral bone DXA, respectively, exist. All three methods demonstrated positive results with accuracy rates even, or greater than previously reported. Moreover, Porto et al. introduced an ANN classifier that could estimate both sex and age through facial photography processing [33]. Furthermore, data obtained from CT scans was used to train algorithms by two scientific teams [29,35]. Both demonstrated positive results and increased accuracy. Fan et al. employed panoramic radiographs to develop DENT-net (a CNN model) used for HI purposes [32]. Finally, Mohammad et al. introduced a dental staging system [21]. 

The PMI estimation is another subject of particular interest, especially when the body is discovered in various stages of putrefaction. Six studies managed to develop ML algorithms that determine PMI from different dataset sources, such as cartilage samples [27], eye images [28], and postmortem gross images of visceral organs [10], while 3 of them focused on postmortem microbiomes [30,31,36]. These studies presented positive results and demonstrated that cartilage might be an ideal matrix for PMI estimation, while images from different organs showed high accuracy. As for the microbiomes they were swabbed from different anatomical sites and the microbial communities were characterized and sequenced. These data were used to train AI algorithms that achieved high accuracy. 

The determination of the COD is naturally of paramount importance for FP. Any means, including the use of AI technologies that can increase the accuracy of COD determination, are therefore considered extremely important. So far, different types of AI algorithms have been trained to detect different death causes, such as fatal head injuries with satisfactory training and testing accuracy [19]. Lin et al. introduced DeepIR which uses data obtained from pulmonary edema fluid spectrochemical analysis to recognize various CODs, such as sudden cardiac death, drowning, intoxication, brain injury, and asphyxiation [20]. Wärmländer et al. developed a k-NN algorithm to estimate the temperature of heat-exposed bone, through portable X-ray fluorescence and measurements [25]. Three studies, included in this systematic review, presented the detection and segmentation of pericardial effusions through CNN, using CT scan results as dataset [26,34,38]. This approach allowed for faster and more accurate diagnosis of pericardial fluid pathology. Concerning drowning cases, two studies [23,24], included in this systematic review, presented algorithms that can identify diatoms and thus contribute to a more evidence-based COD determination. 

It should be noted that so far, available algorithms aim to detect only a fraction of any possible pathology that could represent the COD. No such algorithm that encompasses the whole human pathology yet exists. Evidently, such a task is extremely difficult to achieve.

Nevertheless, future AI applications may affect other forensic fields as well. Such fields may include genetics, image analysis and recognition, and pattern analysis. Concerning forensic genetics, AI may assist in overcoming limitations in techniques such as PCR through statistical software programs [56-62]. It could also help by providing better electropherogram systems that could diminish or eliminate artifacts [63]. Furthermore, when direct comparisons with DNA and/or fingerprint databases are unable to provide a conclusive match, AI may assist by estimating the age of an individual from crime scene biological materials [64-66]. AI might also help by providing better results in DNA extraction and purification methods [61,67-69], while recommendations have already been published for the developmental and internal validation of probabilistic genotyping software [70-76]. 

Image analysis and recognition may be applicable to images obtained either through imaging examinations (e.g. postmortem computed tomography (PMCT) or through digital photography. As already mentioned, many studies included in this systematic review employ datasets from imaging examination data [10,26,29,39]. Future research may provide the ability through analysis of PMCT data, to automatically highlight all important findings and thus aid in the reductions of diagnostic oversights. Relevant research is currently being performed for living patients, with promising results [77-81]. When available, undoubtedly this research may find significant application in FP.

AI use in image analysis and interpretation is currently being researched for living patients in various medical fields, ranging from ophthalmology [82] to histopathology slides [83]. Hopefully, in the future, an AI algorithm will be able to identify postmortem findings (both gross and histopathological), thus limiting the incidence of diagnostic mishaps and facilitating routine morgue operations.

Concerning AI application in pattern analysis, it should be noted that this specific field is probably the most demanding. Pattern analysis may include recording of all forensic findings, their evaluation and subsequent reliable COD proposal, but it may also encompass recognition of specific patterns that may lead to reliable conclusions, especially in crime cases (degree of violence, estimation of fatal injury, etc). Nevertheless, the latter requires the development of extremely large and reliable datasets to train such algorithms, should one day they become available.

Most of the new technologies introduced by the studies included in this systematic review have limitations, such as the requirement for larger sample and data sets, optimization for humans and especially for human cadavers, as well as optimization for specific age groups and nationalities.

To summarize the results extracted by this systematic review, different AI methods have been employed to assist routine forensic work. These algorithms managed to accomplish many tasks with satisfactory accuracy and speed. However, many limitations exist. To overcome these limitations, further research is required, involving creation of larger datasets and updated hardware.

## Conclusions

The AI revolution has brought many changes in the different fields of medicine, including forensic pathology. Different fields of this medical specialty could be assisted by AI and specifically ML algorithms. FA, forensic dentistry, estimation of PMI, and COD are some of these fields. Most of the new technologies introduced by the studies included in this systematic review have limitations. New studies, with larger datasets and upgraded hardware might help to overcome these limitations. To conclude, this systematic review emphasizes the use of AI in FP, by comparing the different technologies that have been used by now and by providing technical information about these technologies. The authors strongly believe that this study could be used as a guide and an inspiration for developing new algorithms, to automate different FP fields. However, it must be clear that AI is not meant to replace the forensic experts but to assist them in their everyday work life.
